# *In vitro* three-dimensional modeling of fallopian tube secretory epithelial cells

**DOI:** 10.1186/1471-2121-14-43

**Published:** 2013-09-27

**Authors:** Kate Lawrenson, Maria Notaridou, Nathan Lee, Elizabeth Benjamin, Ian J Jacobs, Christopher Jones, Simon A Gayther

**Affiliations:** 1 Department of Preventive Medicine, University of Southern California/Keck School of Medicine, 1450 Biggy Street, Los Angeles, California; 2 Gynaecological Cancer Research Laboratories, UCL EGA Institute for Women’s Health, University College London, The Paul O’Gorman Building, Gower Street, London WC1E 6DD, UK; 3 Department of Histopathology, Cancer Institute, University College London, Rockefeller Building, University Street, London WC1E 6JJ, UK; 4 Faculty of Medical and Human Sciences, 1st Floor, Innovation Centre, Core Technology Facility, The University of Manchester, 46 Grafton Street, Manchester M13 9NT, UK

**Keywords:** Fallopian tube secretory epithelial cells, Gene expression microarray, Three-dimensional *in vitro* models, Tissue microenvironment, Ovarian cancer

## Abstract

**Background:**

Fallopian tube secretory epithelial cells (FTSECs) have been implicated as a cell-of-origin for high-grade serous epithelial ovarian cancer. However, there are relatively few *in vitro* models of this tissue type available for use in studies of FTSEC biology and malignant transformation. *In vitro* three-dimensional (3D) cell culture models aim to recreate the architecture and geometry of tissues *in vivo* and restore the complex network of cell-cell/cell-matrix interactions that occur throughout the surface of the cell membrane.

**Results:**

We have established and characterized 3D spheroid culture models of primary FTSECs. FTSEC spheroids contain central cores of hyaline matrix surrounded by mono- or multi-layer epithelial sheets. We found that 3D culturing alters the molecular characteristics of FTSECs compared to 2D cultures of the same cells. Gene expression profiling identified more than a thousand differentially expressed genes between 3D and 2D cultures of the same FTSEC lines. Pathways significantly under-represented in 3D FTSEC cultures were associated with cell cycle progression and DNA replication. This was also reflected in the reduced proliferative indices observed in 3D spheroids stained for the proliferation marker MIB1. Comparisons with gene expression profiles of fresh fallopian tube tissues revealed that 2D FTSEC cultures clustered with follicular phase tubal epithelium, whereas 3D FTSEC cultures clustered with luteal phase samples.

**Conclusions:**

This 3D model of fallopian tube secretory epithelial cells will advance our ability to study the underlying biology and etiology of fallopian tube tissues and the pathogenesis of high-grade serous epithelial ovarian cancer.

## Background

The human fallopian tube is lined by a simple columnar epithelium consisting of both ciliated and secretory epithelial cells. Fallopian tube secretory epithelial cells (FTSECs) are of particular interest given their proposed role as a precursor tissue for high-grade serous epithelial ovarian cancers, which is the most common ovarian cancer histological subtype [1,2]. However, the biology of FTSECs remains poorly understood. This is partly due to difficulties in accessing normal primary FTSECs and in the subsequent development of *in vitro* models of this tissue type. Primary FTSECs have proved challenging to culture, reportedly loosing expression of differentiated markers when propagated *in vitro*. This indicates a cellular plasticity that is strongly influenced by culture conditions [3]. Recent advances in *ex vivo* culture of fallopian epithelia have been achieved by plating the cells onto collagen matrices [4,5]. Under these conditions lineage and differentiation markers are maintained, but unfortunately the cells have an limited capacity for proliferation and cannot be sub-cultured without being immortalized or transformed [6].

Current evidence suggests that FTSECs are a likely origin of high-grade serous epithelial ovarian cancers (HGSOCs) [1,2]. The biological characteristics of the cell-of -origin for different cancers are likely to influence the etiology of the malignant disease [7], including the somatic genetic events that occur during neoplastic development. Gaining a better understanding of the initiation and early stage development of HGSOCs is likely to be of clinical importance. The majority of epithelial ovarian tumors are diagnosed at the late stages (stage III/IV) when 5-year survival rates are only ~30%. In contrast, patients diagnosed with stage I disease have survival rates of over 90%, and are often cured by surgical intervention. The ability to detect HGSOCs in the earliest stages would represent a realistic approach to reducing mortality and a better understanding of the role of FTSECs in the initiation of HGSOCs may be key to the discovery of novel biomarkers associated with early stage disease.

Although the basic functions of all epithelia are the same, there are many fundamental differences in cell morphology, cell function and gene expression across the epithelial cells of different organs. Regardless of cell type, classical cell culture techniques typically involve culturing cells on plastic surfaces that bear limited resemblance to the organs from which the cells originate. Traditional two-dimensional (2D) *in vitro* techniques loose the architecture and geometrical features of tissues *in vivo*, as well as the gradients of nutrients, oxygen, carbon dioxide and other factors that characterize these tissues. Seminal work in three-dimensional (3D) modeling by Bissell and colleagues has shown that culturing normal breast epithelial cells in 3D can induce gland formation, restore cellular polarity and induce upregulated expression of biologically active molecules, thereby simulating the *in vivo* environment [8-10]. Similar approaches have since been used for other epithelial cell types. In most instances, 3D cultures display histological features and differentiated phenotypes that are rarely achieved in 2D cultures [10-12]. The aim of the current study was to establish new 3D models of FTSECs, and to investigate whether 3D FTSEC cultures are more biologically relevant models than monolayer cultures. We developed *in vitro* 3D cultures of FTSECs that mimic features of fallopian tube epithelia *in vivo*; the characteristics of these models suggests that they are suitable for studying both the biology of normal fallopian tube epithelial cells and the early-stage development of HGSOCs.

## Results

### Isolation of fallopian tube secretory epithelial cells

**Figure 1:**
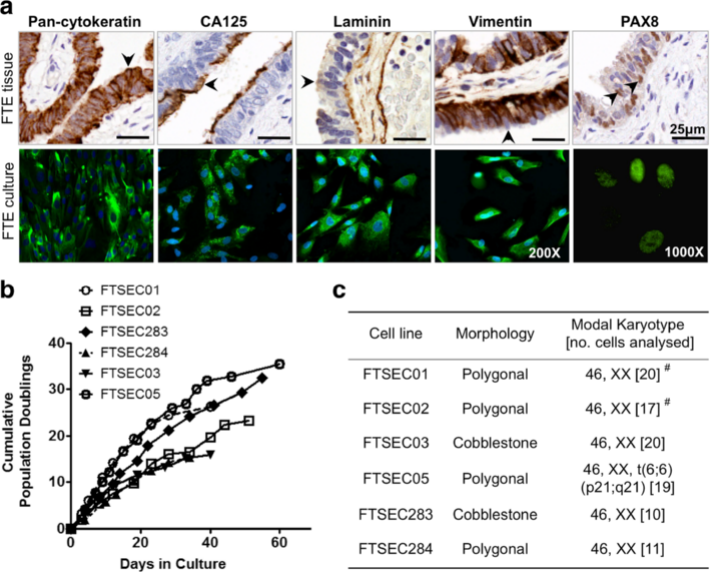
**Characterization of primary FTSEC *in vitro *cultures. (a)** Primary FTSEC cultures maintain expression of markers expressed by fallopian tube secretory epithelia *in vivo* (arrowheads indicate secretory, non-ciliated cells), the FTSEC cultures express cytokeratins, CA125, basal laminin, vimentin and nuclear PAX8. 90-100% of cells stain positive for PAX8 suggesting fallopian tube ciliated epithelial cells do not proliferate *in vitro*. Green stain shows positive staining, nuclei are counterstained with DAPI (blue), except in the case of PAX8. **(b)** Growth curves. FTSECs have a limited *in vitro* lifespan, as is typical for primary normal cells. **(c)** Modal karyotypes for 5/6 primary FTSEC cultures were normal and female (46,XX). ^#^ Two cells or fewer showed unbalanced structural chromosomal rearrangements or numerical abnormalities.

Fallopian tube epithelial cells were isolated from disease-free fallopian tubes of women undergoing partial salpingectomy or total abdominal hysterectomy with bilateral salpingoophorectomy (Table 1). Epithelial cells were harvested from the ampullary regions of fallopian tube samples. Primary cell cultures were confirmed as epithelial by immunofluorescent staining to analyze expression of cytokeratin (Figure 1a). Two of five FTSEC cultures also expressed the gynecological epithelial cell marker CA125. The absence of stromal contaminants was shown by absence of staining for Von Willenbrand Factor VIII, which is expressed by endothelial cells, and the fibroblastic marker fibroblast surface protein (data not shown). Almost all cells in FTSEC cultures expressed the lineage-specific marker PAX8 in the nucleus (Figure 1a), indicating that the cell culture protocol enriched for fallopian tube secretory epithelial cells (FTSECs) [1,13]. FTSECs also expressed vimentin and laminin (Figure 1a). FTSECs could be successfully subcultured but had a limited lifespan in culture, which is typical of primary cells. Primary FTSECs proliferated for 34–60 days (equating to 10–16 passages in culture) (Figure 1b) at which point cells acquired senescent morphologies and expressed senescence-associated β-galactosidase (data not shown). The modal karyotype for 5/6 cell lines was 46,XX (Figure 1c); one cell line (FTSEC05) displayed an abnormal karyotype of 46, XX, t(6;6) (p21;q21) in all of the 19 cells analyzed, and was therefore excluded from further analyses.

**Table 1:** Patient information

**Cell line**	**Patient age**	**Menopausal status**	**Clinical data**	**Histopathological diagnosis**	**Histopathology report – ovaries and fallopian tubes**
**FTE01**	51	NK	Mucinous cyst on the left ovary	Benign follicular and epithelial inclusion cysts. Benign serous cystadenofibroma.	Fallopian tubes normal
**FTE02**	50	NK	Benign fibrosis	Leiomyomas and folicular ovarian cysts	Mild chronic salpingitis in one tube but no evidence of malignancy
**FTE03**	38	Pre	Fibroid. Adenomyosis	Secretory endometrium and benign leiomyoma	Fallopian tubes normal
**FTE05**	54	Post	Complex hyperplasia with atypia	Autolysed endometrium. No evidence of invasive carcinoma. Benign leiomyomas.	Post-menopausal features present in both ovaries. Fallopian tubes normal.
**FTE283**	65	Post	Grade 2–3 endometrial cancer	Grade 2 endometrioid adenocarcinoma of the endometrium, with >50% myometrial invasion	Post-menopausal features present in both ovaries. Fallopian tubes normal.
**FTE284**	60	Post	History proven endometrial cancer	Grade 2 endometrioid adenocarcinoma of the endometrium	Fallopian tubes normal

FTSEC cell lines were collected from patients aged between 38 and 65 years of age. For all patients, fallopian tube tissues were confirmed to be histologically normal. For the pre-menopausal patient the phase of the menstrual cycle was not known. NK = not known.

### Three-dimensional culturing of FTSECs

**Figure 2:**
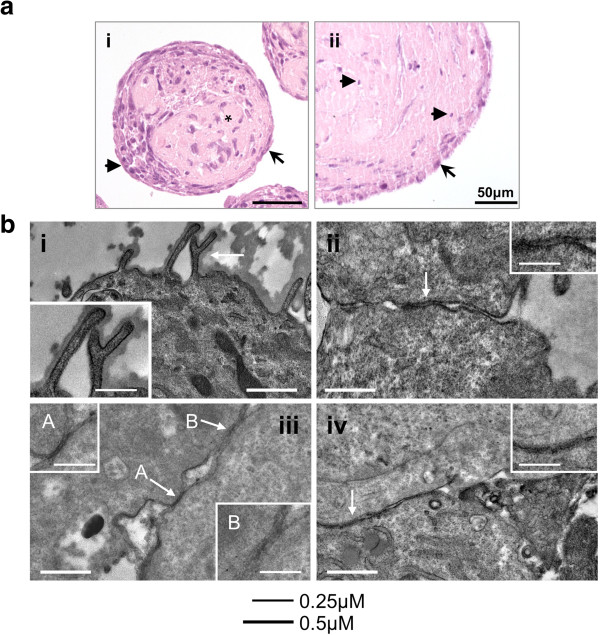
**Histological analysis of 3D FTSEC spheroids. (a)** Three dimensional spheroid cultures stained by hematoxylin and eosin. At (i) 15 days, the core of the spheroids contains abundant matrix material (*), covered by an epithelial cell monolayer (arrow) or cellular cap structure (arrowhead). (ii) After 40 days no viable cells remain within the core of the spheroids. A viable monolayer covers the surface of the spheroid (arrow), degenerate nuclear debris can be seen within the matrix core (arrowhead). **(b)** Electron microscopy of FTSEC spheroids. Features of epithelial cells *in vivo* are detected in 3D cultured FTSECs including (i) microvilli and cell-cell junction complexes including (ii,iii) tight junctions (white arrows and inset) and (iv) adherens junctions (white arrows and inset). Electron microscopy.

**Figure 3:**
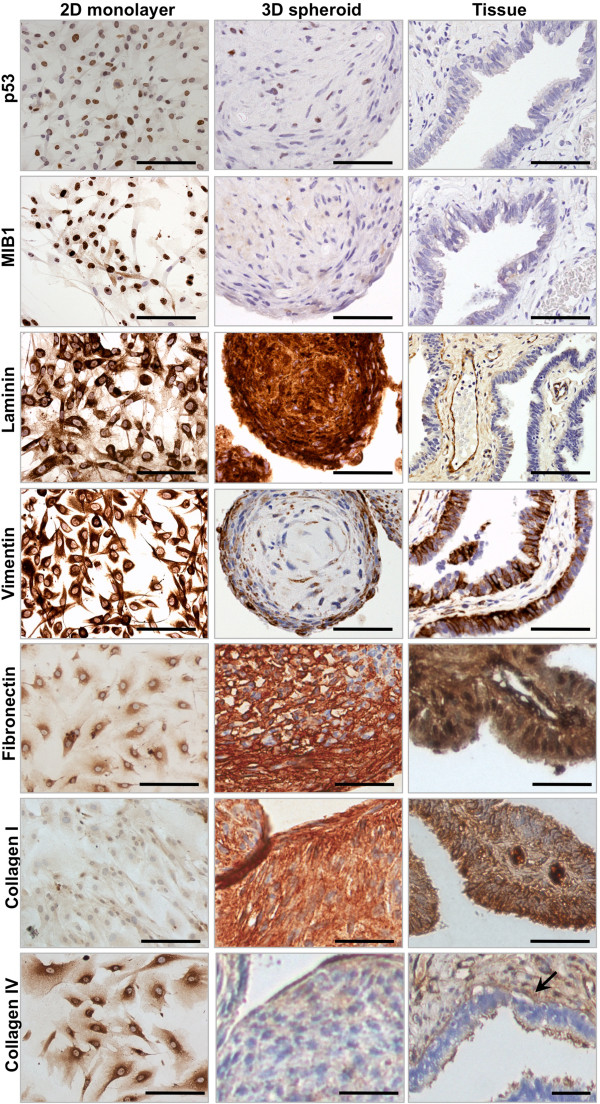
**Immunohistochemical staining of 2D and 3D cultured FTSECs and primary fallopian tube tissue.** Immunohistochemical staining of p53 (clone Do7) and a proliferation marker, MIB1, shows high expression of both markers in 2D cultured cells but low expression in 3D FTSEC cultures (at day 15), and fallopian tube epithelia *in vivo*. Laminin and vimentin are expressed in 2D cultured cells, 3D cultured cells and *in vivo.* Expression of fibronectin and collagen I was lower in 2D cultured cells than 3D cultures and fallopian tube epithelia in fresh tissue specimens. Collagen IV is predominantly expressed in the basal lamina (arrow) and stroma of fallopian tubes but was expressed at high levels by 2D cultured cells. Expression of this marker was low/absent in 3D cultured cells. * Fibronectin, collagen I and collagen IV were also examined in FTSEC01, expression patterns were highly similar to data for FTSEC283 and FTSEC03. Expression of collagen IV was restricted to the basal lamina and stroma. None of the markers examined were differentially expressed by ciliated and secretory fallopian tube epithelial cells *in vivo.* As our cell isolates were predominantly PAX8 positive (Figure 1), we do not expect ciliated cells to be present in the FTSEC spheroid cultures. Brown stain denotes positive antigen detection, cells are counterstained with eosin (blue). Light microscopy. Scale bars = 100 μm.

Three-dimensional (3D) cultures of FTSECs were established by inoculating cells into polyHEMA-coated tissue culture plastics as previously described for normal and transformed ovarian epithelial cells [14-16]. Within 24 hours of culture, FTSECs aggregated and spontaneously formed multicellular spheroids. After 14 days, FTSEC spheroids were fixed, paraffin-embedded and sectioned, and the histological features examined by hematoxylin and eosin (H&E) staining. All five primary lines grew as spheroids and revealed a similar cellular architecture. A monolayer of epithelial-like cells typically surrounded each spheroid and in some instances there was also multi-layering of the epithelium. FTSEC spheroids commonly displayed a crescent-shaped cellular cap structure, which we have previously described for primary normal ovarian epithelial cell cultures in 3D [14]. The centre of the spheroids comprised a hyaline matrix that resembled the extracellular matrix present in the *in vivo* tissue in composition (Figures 2 and 3). We observed some viable cells amongst abundant karyorrhectic debri (nuclear dust) within the matrix core of the spheroids. Many of the viable cells within spheroid cores (but not cells on the periphery) exhibited nuclear and cellular pleomorphism, suggesting these cells undergo apoptosis and degenerate (Figure 2a (ii)). In doing so, these cells contribute to the matrix material that makes up the structure of the core of FTSEC spheroids. The internal structure and sub-cellular features of three-dimensional spheroid cultures of FTSECs, examined by transmission electron microscopy revealed features of epithelial cells, including microvilli (Figure 2b (i)), tight junctions (Figure 2b (ii & iii)) and adherens junctions (Figure 2b (iv)).

We compared molecular features between 2D and 3D FTSEC cultures using immunohistochemistry for series of biomarkers either known to be expressed in normal fallopian tube epithelia or relevant to the biology of FTSECs in serous carcinogenesis (Figure 3, Table 2). FTSECS are not highly proliferative *in vivo*, but have high proliferative indices when cultured as a monolayer (Figure 1b and Figure 3). MIB1, which is expressed during G1, S, G2 and M phases of the cell cycle, and p53, which is expressed at the G2/M cell cycle checkpoint both showed marked reductions in expression in 3D cultured FTSECs compared to 2D cultures, suggesting that FTSECs are less proliferative in 3D compared to 2D (Figure 3 and Table 2). This is consistent with the expression of these markers *in vivo*. PAX8 and E-Cadherin showed no reproducible changes in expression in 2D compared to 3D cultures. Vimentin showed higher expression in 2D cultured cells and in primary tissue, but showed a modest reduction in expression in 3D for all cell lines examined. The basement membrane protein laminin was expressed at high levels in both 2D and 3D cultures. Fibronectin and collagen I were expressed at high levels by epithelial cells of the fallopian tube; these markers were expressed at low levels in 2D FTSEC cultures and were then upregulated in 3D. Collagen IV expression was restricted to the basal lamina and stroma of human fallopian tube tissues but was ectopically expressed at high levels in 2D cultures and then reduced in 3D cultures, which is more reflective of human tissues. One cell line (FTE283) showed increased expression of mucins (mucin 1 and mucin 16, also known as CA125) in 3D cultures, but for the other cell lines these mucins were either expressed at low levels (in 2D cultures) or not all (in 3D cultures).

**Table 2:** Summary of immunohistochemical staining of FTSEC spheroids

	**FTE02**		**FTE03**		**FTE283**		**FTE284**		**TISSUE**	
***Marker***	**2D**	**3D**	**2D**	**3D**	**2D**	**3D**	**2D**	**3D**	**FTE03**	**FTE04**
AE1:AE3	++	+/−	+++	+	+++	++++	++++	++	++++	++++
VIMENTIN	+++	++	++++	+++	++++	+++	++++	+++	++++	+++
LAMININ	++++	++++	++++	++++	++++	++++	++++	++++	++	++
MUC-1	++	+/−	+	-	+	+++	++	-	++++	+++
CA125	+	-	+	-	+/−	++	++	-	+++	+++
PAX8	+	-	++	+/−	+++	+++	++	+/−	+++	++
TP53 (Do7)	+++	+/−	+++	+	++	+	+++	+/−	+	+/−
MIB-1 (%)	70%	<5%	70%	<5%	70%	<5%	50%	<5%	<5%	<5%

Immunohistochemistry was performed on five FTSEC cultures grown as 2D monolayers and as 3D spheroids, and compared to two fresh tissue specimens. Staining of pan-cytokeratin (AE1:AE3), mucin-1, CA125, PAX8 and E-Cadherin showed no significant changes in 2D versus 3D cultured cells. Laminin showed high levels of expression in all samples and expression along the basement membrane of fallopian tube epithelial cells *in vivo*. TP53 and MIB1 expression were high in 2D cultured cells, but low in 3D cultured cells and *in vivo*.

### *Whole transcriptome analyses of* FTSECs

**Figure 4:**
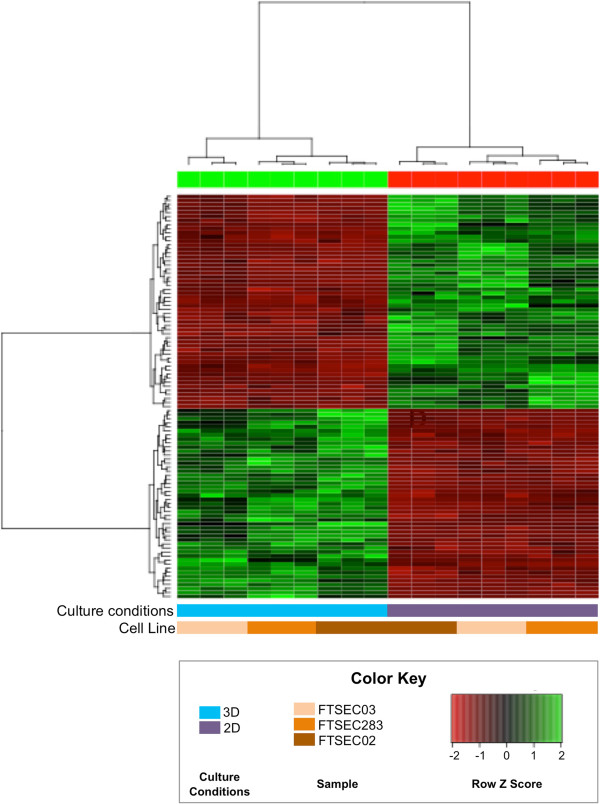
**Gene expression microarray analysis of 2D and 3D cultured FTSECs.** Hierarchical clustering of transcriptomic profiles of the 100 genes showing the most significant changes in expression between three 2D and 3D cultured FTSEC primary lines. Three independent samples were prepared for each cell line and each culture condition, each sample represents an individual microarray, technical replicates are shown. Cell lines cluster according to culture conditions, not patient.

**Figure 5:**
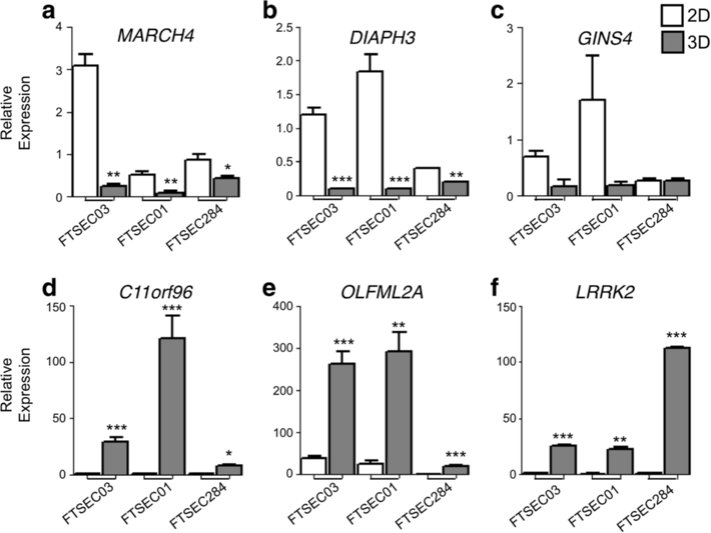
**Validation of genes identified through expression microarray profiling of 2D and 3D cultured FTSECs.** The most significantly changing genes were validated by TaqMan qPCR in independent 2D and 3D cultures of FTSEC03, as well as in two additional cell lines from different patients (FTSEC01 and FTSEC284). Genes downregulated in 3D cultured cells **(a)***MARCH4* and **(b)***DIAPH3* were significantly downregulated in all 3 cell lines tested with the exception. **(c)** A similar trend was observed for *GINS4* although this did not reach statistical significance. Genes upregulated in 3D cultured cells **(d)***C11orf96*, **(e)***OLFM2A* and **(f)***LRRK2* were all upregulated in all 3 samples following a transition to a 3D microenvironment. * *P* > 0.05, ** *P* > 0.01, *** *P* > 0.001, two-tailed paired Student’s T-tests were used to compare 2D and 3D cultured cells for each cell line individually.

We profiled the genes and pathways differentially expressed when FTSECs transition from a 2D to 3D microenvironment. Three FTSEC lines were cultured as 2D monolayers and 3D spheroids for 4 days and whole transcriptome profiling performed using the Illumina HT12 beadchip microarrays. In total, 1005 probes were differentially expressed between 2D and 3D cultures (SAM analysis, FDR = 5.80×10^-5^, two-class unpaired test, δ = 5.16). Figure 4 shows a heatmap of the top 100 significantly changing genes between 2D and 3D cultures (also listed in Additional file 1: Table S1). Among the most significantly down-regulated genes were those coding for membrane proteins (*MARCH4*, fold change (FC) 0.065; *TMEM106C*, FC = 0.22), DNA repair proteins (*RAD51C*, represented by 2 probes showing fold changes of 0.44 and 0.46) and Rho signaling proteins (*DIAPH3*, FC = 0.13; *CDC42EP3*, FC = 0.16). Genes that were up-regulated in 3D cultured cells included those coding for ATP-binding cassette transporters (*ABCC3*, FC = 4.19; *ABCA2*, FC = 3.24) and trans-membrane proteins (*TMTC1*, FC = 1.97; *TMCO3*, FC = 2.05; TMEM117, FC = 2.19) (Table 3). Hierarchical clustering of Elucidean distances between samples showed that the differences were greater between 2D and 3D culture conditions than for FTSECs from different patients (Figure 4). The three most significantly up- and down-regulated genes were validated by qPCR (Additional file 2: Figure S1 and Figure 5). *MARCH4* and *DIAPH3* were significantly downregulated in 3D cultured cells compared to 2D cultures (*P* > 0.05, Figure 5a,b). *GINS4* showed a similar trend although changes in expression in 2D versus 3D were not statistically significant (Figure 5c). *C11orf96, OLFM2A* and *LRRK2* were consistently overexpressed in 3D cultured cells compared to the same cells cultured in 2D (Figure 5d-f, *P* > 0.05).

**Table 3:** Analysis of gene ontology terms over-represented in 3D cultured cells

**GO terms over-represented in genes down-regulated in 3D cultured cells**				
**Term**	**Exp count**	**Count**	**Size**	**Adjusted P-value**
Organelle fission	11.106	61	321	2.00E-25
Cell division	8.987	50	266	7.74E-21
Mitotic prometaphase	2.872	30	83	2.50E-20
Cell cycle process	10.267	44	347	7.74E-14
Cell cycle checkpoint	2.962	23	87	3.72E-12
Interphase	11.522	41	333	4.02E-10
Chromosome segregation	2.835	20	84	1.14E-09
Regulation of cell cycle process	8.241	33	245	2.28E-09
Cellular component organization or biogenesis	104.766	163	3028	9.59E-09
Cellular component organization at cellular level	73.542	125	2225	1.97E-08
DNA replication	5.364	25	157	2.37E-08
DNA strand elongation involved in DNA replication	1.073	12	31	3.95E-08
Mitotic cell cycle	5.122	24	175	4.65E-08
Negative regulation of cell cycle	11.162	35	326	2.89E-07
DNA metabolic process	4.759	21	159	1.64E-06
Microtubule anchoring	0.830	9	24	8.20E-06
Chromosome organization	15.877	39	475	2.38E-05
Cellular response to stress	29.513	59	853	2.38E-05
Mitosis	4.269	18	137	3.02E-05
Regulation of microtubule-based process	2.284	13	66	3.94E-05
Telomere maintenance via recombination	0.796	8	23	6.95E-05
DNA recombination	3.716	16	111	8.96E-05
Telomere maintenance via semi-conservative replication	0.830	8	24	9.23E-05
Double-strand break repair via homologous recombination	1.419	10	41	9.61E-05
DNA repair	6.807	22	206	1.17E-04
Mitotic spindle organization	0.865	8	25	1.17E-04
Microtubule-based process	4.335	17	132	1.28E-04
Chromosome localization	0.657	7	19	1.74E-04
Regulation of transcription involved in G1/S phase of mitotic cell cycle	0.692	7	20	2.50E-04
CenH3-containing nucleosome assembly at centromere	0.727	7	21	3.41E-04
DNA replication-independent nucleosome organization	0.727	7	21	3.41E-04
DNA packaging	2.857	13	84	3.56E-04
S phase of mitotic cell cycle	4.325	16	125	4.55E-04
Nucleotide-excision repair, DNA gap filling	0.623	6	18	0.002
ATP-dependent chromatin remodeling	0.934	7	27	0.002
Nucleosome assembly	2.491	11	72	0.002
Microtubule-based movement	3.460	13	100	0.003
G1/S transition of mitotic cell cycle	4.693	15	138	0.004
Anaphase-promoting complex-dependent proteasomal ubiquitin-dependent protein catabolic process	2.733	11	79	0.005
Transcription-coupled nucleotide-excision repair	1.488	8	43	0.006
Mitotic sister chromatid segregation	0.510	5	15	0.006
Establishment of organelle localization	2.371	10	69	0.007
Cellular component assembly	34.876	57	1008	0.007
Positive regulation of cell cycle cytokinesis	0.311	4	9	0.009
Mitotic metaphase	0.138	3	4	0.009
Spindle organization	0.605	5	18	0.013
Mitochondrial translation	0.346	4	10	0.013
DNA-dependent DNA replication initiation	0.934	6	27	0.013
G2/M transition DNA damage checkpoint	0.934	6	27	0.013
Maintenance of protein location	2.630	10	76	0.013
G2/M transition of mitotic cell cycle	3.646	12	106	0.013
Phosphatidylinositol-mediated signaling	2.180	9	63	0.014
Maintenance of location in cell	2.664	10	77	0.014
Spliceosomal snRNP assembly	0.969	6	28	0.015
Meiotic cell cycle	3.737	12	108	0.015
Microtubule depolymerization	0.657	5	19	0.016
Telomere organization	1.799	8	52	0.016
Mitotic chromosome condensation	0.381	4	11	0.016
Regulation of attachment of spindle microtubules to kinetochore	0.173	3	5	0.016
Spindle assembly	1.001	6	29	0.016
RNA splicing, via transesterification reactions	6.747	17	195	0.017
Cytoskeleton organization	12.037	25	356	0.017
Macromolecular complex subunit organization	3.893	12	119	0.020
Negative regulation of organelle organization	4.048	12	117	0.028
Attachment of spindle microtubules to kinetochore	0.205	3	6	0.028
Mitotic metaphase plate congression	0.450	4	13	0.029
Response to ionizing radiation	3.010	10	87	0.030
Regulation of cyclin-dependent protein kinase activity	2.526	9	73	0.032
Regulation of mitosis	1.165	6	34	0.033
RNA processing	20.137	35	582	0.034
Metaphase plate congression	0.069	2	2	0.039
Aspartate biosynthetic process	0.069	2	2	0.039
Glutamate catabolic process to aspartate	0.069	2	2	0.039
Glutamate catabolic process to 2-oxoglutarate	0.069	2	2	0.039
Regulation of hippo signaling cascade	0.069	2	2	0.039
Negative regulation of mitotic recombination	0.069	2	2	0.039
Deoxyribonucleotide biosynthetic process	0.242	3	7	0.040
Kinetochore assembly	0.242	3	7	0.040
Protein K6-linked ubiquitination	0.242	3	7	0.040
M phase of mitotic cell cycle	0.239	3	8	0.041
**GO terms over-represented in genes upregulated in 3D cultured cells**				
**Term**	**Exp count**	**Count**	**Size**	**Adjusted P-value**
Positive regulation of transcription from RNA polymerase II promoter	9.063	26	362	0.003

80 terms were over-represented in the genes that were underexpressed in 3D and 1 GO term was significantly over-represented in genes upregulated when FTSECs were transitioned to a 3D microenvironment. The majority of these terms were associated with cell cycle progression, mitosis and DNA replication.

We performed gene ontology (GO) analyses using the top 1005 probes representing 821 unique Entrez identifiers. For a sub-set of 354 identifiers that were significantly downregulated in 3D cultures, 80 GO terms were significantly over-represented; 75% of these were associated with cell division, mitosis, telomere maintenance, DNA replication and repair (Table 3). The most significantly over-represented term was organelle fission (*P* = 2.00×10^-25^). Positive regulation of transcription from RNA polymerase II promotor was the only GO term significantly over-represented in the 467 identifiers that were overexpressed in 3D cultures (Table 3), which is likely to reflect the widespread changes in gene expression observed when FTSECs transition from a 2D to 3D microenvironment. No GO terms were found to be under-represented in the 1005 probes that significantly different in the comparison of 2D and 3D FTSEC cultures.

**Figure 6:**
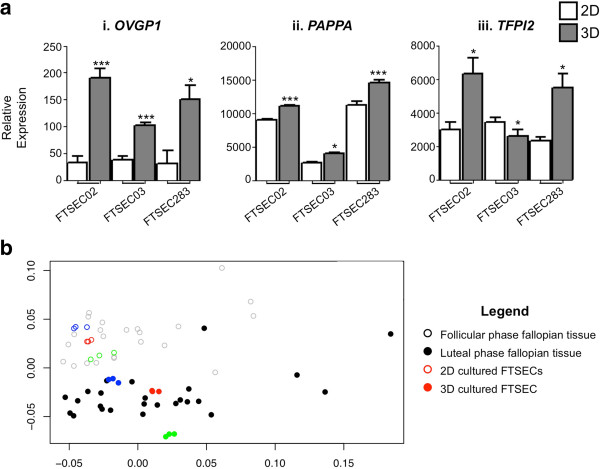
**FTSEC biomarker expression in 2D and 3D and cluster analysis comparing genome-wide transcriptomic profiles of 2D and 3D cultured FTSECs to follicular and luteal phase fallopian tube epithelium. (a)** Expression of FTSEC biomarkers is typically higher in 3D than in 2D (i) oviductal glycoprotein 1, (ii) pregnanacy-associated plasma protein A and (iii) tissue factor pathway inhibitor 2. **(b)** Euclidean Pearson clustering analyses reveal that 2D cultured FTSECs cluster with follicular phase fallopian tube epithelium, whereas 3D cultured fallopian tube epithelial cells cluster with luteal phase tubal epithelium. Each point on the graph indicates an individual microarray profile, technical replicates of cultured cells are shown by colored circles, open circles denoting 2D cultured cells and closed circles denoting 3D cultured FTSECs. Black open circles indicate follicular phase fallopian tube epithelial samples, closed circles indicate luteal phase fallopian tube epithelium. Each point represents an individual patient. All patients from Georges *et al*. and Tone *et al*. [17,18] datasets are shown.

We took two approaches to examine whether 3D culturing of FTSECs affects functional differentiation. Firstly we examined expression of genes that encode proteins known to be secreted by FTSECs *in vivo*: oviduct specific glycoprotein 1 (*OVGP1*), pregnancy-associated plasma protein A (*PAPPA*) and tissue factor pathway inhibitor 2 (*TFPI2*, also known as placental protein 5). *OVGP1* and *PAPPA* were significantly upregulated by all 3 FTSEC cultures following the transition to 3D (*P* > 0.05, Figure 6a). *TFPI2* was also significantly upregulated in 2/3 3D cultures (*P* > 0.05). Secondly, we compared gene expression profiles of 2D and 3D FTSEC cultures with profiles of fresh human fallopian tube tissue specimens. Datasets representing fallopian tube epithelial tissues harvested at different points of the menstrual cycle were selected (Tone *et al.* and George *et al.*[17,18]). We used cluster analysis to examine the similarities between global transcriptomic profiles of 2D cultured FTSECs, 3D cultured FTSECs, luteal phase fallopian tube epithelial cells and follicular phase fallopian tube epithelial cells. Regardless of the clustering method used (maximum or Euclidean distance, calculated from either the Pearson or Spearman correlation coefficients), profiles from 2D cultures clustered with fallopian tube epithelial tissues collected during the follicular phase of the menstrual cycle, whilst 3D-cultured cells consistently clustered with luteal phase fallopian tube epithelium (Figure 6b and Additional file 3: Figure S2).

## Discussion

Here, we describe a novel approach to model normal primary fallopian tube secretory epithelial cells (FTSECs) in an *in vitro* three-dimensional (3D) spheroid system. Culturing FTSECs as spheroids restores the 3D architecture of the tissue *in vivo*, as well as gradients of nutrients, oxygen, carbon dioxide and other macromolecules. We observed molecular and cellular features of FTSECs cultured in 3D more closely resembled fresh FTSEC tissue samples than monolayer cultured fallopian tube secretory epithelial cells. One striking change associated with the transition to 3D was the reduced proliferation rate of cells in 3D compared to 2D, as demonstrated by MIB1 and p53 staining. Cells in 3D were less proliferative which was also reflected in the changing patterns of gene expression following transition from 2D to 3D. This is consistent with a previous study of normal ovarian surface epithelial cells cultured in 3D cultures [14], and is also true for normal breast cells [19]. Since proliferation of the fallopian tube mucosa occurs in pre-malignant or malignant lesions [20], these data suggest that these 3D models more closely reflect the quiescent status of normal FTSECs *in vivo* and are more biologically relevant models of normal FTSECs than 2D monolayers for studying normal fallopian tube biology and tumorigenesis. Furthermore, 3D culturing enhanced the production of secretory products by FTSECs. Oviduct specific glycoprotein 1 (*OVGP1*), also known as mucin 9, is normally secreted by non-cilated tubal epithelia and improves *in vitro* fertilization rates by reducing polyspermy and increasing blastocyst formation rates [21,22]. We found *OVGP1* to be upregulated ~2-4 fold in FTSECs cultured in 3D. Similarly, a second glycoprotein, pregnancy associated plasma protein A (*PAPPA*) was also significantly upregulated in 3D. Increased expression of these bioactive glycoprotein molecules suggests FTSECs grown in 3D have enhanced functional differentiation compared to their 2D counterparts.

We compared global expression profiles of 2D and 3D cultured cells with biomarker expression in primary fresh fallopian tube tissue samples. We showed that gene profiles in 2D cultured cells cluster with follicular phase fallopian epithelial tissue, whereas 3D cultured cells cluster with luteal phase fallopian tube samples. This result may also be driven by the proliferative signature of the 2D cultured cells, as the follicular phase of the menstrual cycle is the proliferative phase, when raised levels of estradiol stimulate proliferation of the epithelia lining the endometrium and fallopian tube [23]. We found that gene expression profiles of 3D cultured FTSECs cluster with those of luteal phase fallopian tube tissues. This phase of the cell cycle is the secretory phase, which may indicate a commitment to secretory differentiation FTSECs cultured in 3D. Consistent with this, we observed upregulation of an secreted proteins as well as an FTSEC marker (PAX8) when one FTSEC line was cultured in 3D. These data strongly suggest that culturing in 3D enhances functional differentiation of FTSECs to a secretory phenotype.

Previous studies have reported culture of human fallopian tube epithelia *ex vivo*, on collagen gel and alginate matrices [4,5]. These models have significantly advanced our ability to model human and murine polarized fallopian tube epithelia *in vitro*. However, one limitation of *ex vivo* models is the restricted ability to sub-culture the cells. Using a growth factor rich media we were able to subculture the fallopian tube epithelial cells we isolated. We then selected a spheroid culture method to establish 3D cultures because this approach offers flexibility for downstream molecular analysis, and can be scaled up or down to perform high-throughput molecular screening or large-scale mass cultures. Although we did not supply matrix proteins in the cultures, fallopian tube secretory epithelial cells produced a matrix of which laminin was a major component. Laminin is the major protein in the basal lamina, the aspect of the basement membrane to which epithelial cells are adhered *in vivo* via integrin-mediated interactions. We hypothesize that altered cell-matrix interactions may contribute to the altered gene expression patterns we observed. While the 3D FTSEC cultures presented here do not recreate the complex convoluted architecture of the lumen of a fallopian tube *in vivo*, in FTSEC spheroids the epithelial cell-basement membrane interaction is restored. We observed that the outer surface of the spheroid is reminiscent of the lumen of the fallopian tube in that cells are in contact with other mucosal epithelia throughout the lateral domains of the cell, and basal domains of the cells are in contact with a basement membrane-type matrix. In contrast, cells trapped within the spheroid cores are surrounded by matrix, which is an ectopic microenvironment for normal epithelial cells. We hypothesize that this may induce programmed cell death, resulting in the high frequency of apoptotic cell debris observed within the cores but not at the periphery of FTSEC spheroids. Alternatively the physiological conditions within spheroids could have contributed to the changes survival of cells at the centre of the multicellular aggregates, since mathematical modeling suggests that deficiencies in ATP [24], glucose, hydrogen and oxygen [25] may all induce necrotic cell death of cells within spheroid cores.

Many key cellular processes are now known to be differently regulated between 2D and 3D cultures, and various factors can induce differential gene expression in 3D, including altered cell-cell and/or cell-matrix communications, nutrient and oxygen gradients, and reduced rates of proliferation. We propose that the 3D models are more biologically relevant tools of FTSECs than traditional 2D monolayers with which to study fallopian tube epithelial cell biology and pathogenesis. Perhaps the greatest potential for clinical impact of these models will come from their use in studies of tumor initiation. This has become particularly significant since it was established recently that the epithelia lining of the fallopian tube likely represents the cell of origin for a proportion of HGSOCs. HGSOCs bear morphological resemblance to Müllerian epithelia and over 80% of this tumor type overexpress PAX8 [26], an FTSEC marker that can be used to distinguish ovarian serous tumors from other, morphologically similar neoplasms [27,28]. We identified additional FTSEC biomarkers that represent novel candidate HGSOC biomarkers. These include *LRRK2*, a gene that encodes a kinase involved in Parkinsons Disease [29,30]. *LRRK2* has not previously been implicated in ovarian cancer development but analyses of The Cancer Genome Atlas (TCGA) data suggests ~3% of primary HGSOCs harbor somatic mutations in this gene [31-33]*.* Other novel FTSEC biomarkers that are overexpressed in HGSOCs include *CELSR3,* an atypical cadherin; *ABCC3*, an ABC transport protein implicated in drug resistance [34]; and *CTHRC1,* a secreted protein shown to be a candidate biomarker for breast and pancreatic cancer [35,36]*.* Analyses of primary HGSOC specimens and sera collected from ovarian cancer patients will be required to determine whether any of these novel biomarkers have clinical utility in the early detection of HGSOC.

While it is now widely accepted that a proportion of HGSOCS originate in the fallopian tube, the early stages of disease development are poorly understood and many questions remain to be answered. Reports show differences in the proportions of ciliated and secretory epithelial cells, marker expression and hormone responsiveness between the epithelia found in fimbrial and ampullary regions of the fallopian tube [23,37,38]. However, as yet we do not yet know why FTSECs in the fimbrial region of the fallopian tube are more prone to neoplastic transformation. One hypothesis is that the proximity to the mitogenic environment of the ovarian stroma may influence the phenotype of fimbrial FTSECs. Alternatively the region of transition between FTSECs and ovarian mesothelial-type epithelial cells is inherently more prone to neoplastic transformation. In the future, these 3D models of FTSEC transformation that incorporate common somatic genetic alterations characteristic of HGSOC (*BRCA1*, *p53*[31]) or even recently discovered susceptibility alleles that confer low-risk of EOC in the general population [39-45] will be vital tools in answering some of the key questions regarding EOC initiation and development.

## Conclusion

In conclusion we have developed a novel 3D *in vitro* culture model of fallopian tube secretory cells that represent a precursor tissue of high-grade serous ovarian cancer. The greatest potential clinical use for these models is likely to come from molecular and phenotypic studies of the initiation and early stage development of ovarian cancer leading to the discovery of novel biomarkers for early stage disease detection. These models may also have applications beyond the study of ovarian carcinogenesis, for example for studying the interactions between the fallopian tube epithelium and oocytes or zygotes. Co-culture of fallopian tube epithelial cells has been shown to promote the *in vitro* development of embryos. In future, novel 3D co-culture methodologies, in which glycoprotein secretion is enhanced, may improve *in vitro* embryogenesis. Models of benign fallopian tube diseases that are commonly associated with female infertility, such as salpingitis and pelvic inflammatory disease, are also few in number; but the models we describe here could be used to mimic such conditions *in vitro* and help to improve their diagnosis and treatment. Ultimately, it is hoped that these models will lead to much needed insights into the biology and pathogenesis of fallopian secretory epithelial cells and that this knowledge with be invaluable in increasing our ability to diagnose and treat benign and malignant disease arising in the fallopian tubes.

## Methods

### Tissue collection and cell culture

Patients scheduled to undergo surgical procedures for benign gynecological conditions (including fibroids, polyps) or total abdominal hysterectomies for endometrial cancer provided informed written consent, prior to surgery, agreeing to participate in the study. This study was performed with permission of the UCL Institutional Ethics Committee. Fallopian tubes were inspected by the operating surgeon and a gynecological pathologist and confirmed to be free of malignancy. The distal ampullary region of the fallopian tube was isolated and dissected open to reveal the lumen. Epithelial cells were harvested by gentle brushing with a sterile cytobrush. All FTSEC cell cultures were maintained in MCDB105:Medium 199 (mixed in a 1:1 ratio) supplemented with 15% fetal bovine serum (Hyclone), 10 ng/ml epidermal growth factor, 0.5 mg/ml hydrocortisone, 5 mg/ml insulin, and 34 mg protein/ml bovine pituitary extract, (all Sigma, St Louis, MO, USA). For growth curves 1 × 10^5^ cells were plated in triplicate. Cultures were passaged and population doublings (PD) calculated using the following formula:

PD = log (total cell number at each passage/initial cell number)/log2.

For analysis of cellular karyotype, cells were taken at a low passage and seeded at low density in a 25 cm^2^ flask. The karyotypes were analysed by a certified clinical cytogeneticist (TDL genetics, London, UK). The number of chromosomes as well as their length, the position of the centromeres, banding pattern and any other physical characteristics were commented on to give a detailed description of any abnormalities.

### Immunofluorescent cytochemistry

Cell monolayers were grown on glass coverslips to of ~80% confluency. Cells were washed with ice cold PBS-Ag (PBS with 0.3% fish skin gelatin, Sigma) and fixed for 10 min in 3% paraformaldehyde, then re-washed with PBS-Ag. Cells were permeabilized with 0.3%v/v Triton X-100 in PBS-Ag, rinsed twice again and blocked with goat serum (Invitrogen, Carlsbad, CA, USA) for 30 min. Primary antibodies were diluted 1:1000 in PBSAg and applied to the cells for 1 hour. After rinsing the antibody, secondary was applied for 20 min in the dark, Alexa Fluor 488-coupled secondary or antibodies (Invitrogen) were used for antigen detection. The coverslips were then rinsed and transferred to labelled slides to add DAPI stain for nuclear staining. The slides were viewed under an Olympus BX64 fluorescence microscope (Olympus, Tokyo, Japan) and images were captured and analyzed using Cytovision Genus 3.6 Software (Leica, Wetzlar, Germany).

### Three-dimensional cell culture and immunohistochemistry

Tissue culture vessels were twice coated with a 1.5% of poly-2-hydroxyethyl methacrylate (polyHEMA, Sigma) solution in 95% ethanol, and allowed to dry. Before use, polyHEMA coated plates were washed with sterile PBS. Cells were trypsinised and counted, and 1×10^5^ cells plated into polyHEMA coated P100 dishes in 25 mls complete medium. To fix the 3D cultures, spheroids were collected into a 50 ml falcon tube washed twce in PBS and fixed for 30 mins in neutal-buffered formalin (VWR, West Chester, PA, USA). Fixed 3D cultures were then processed into paraffin blocks, sectioned and stained by immunohistochemistry at UCL Advanced Diagnostics immunocytochemistry service laboratory (UCL, London) and at the Translational Pathology Core Facility at UCLA, Los Angeles, California. Staining was performed using standard immunohistochemical staining techniques with the following antibodies collagen type I (Santa Cruz Biotechnology, Santa Cruz, CA, USA), collagen type 4 (Abcam, Cambridge, MA, USA), laminin (Dako, Glostrup, Denmark), pan-cytokeratin (clone AE1/AE3, Dako), p53 (Do7, Dako) and MIB1 (Dako).

### Transmission electron microscopy

FTSECs were grown as 3D spheroids for 4 days, after which cells were harvested by centrifugation and the culture media aspirated. Spheroids were washed with PBS and fixed with ½ strength Karnovsky’s Fixative overnight at 4°C. Spheroids were then rinsed in 0.1 M Cacodylate Buffer for 10mins, post-fixed in 2% Osmium Tetroxide for 1 hour, then rinsed again in 0.1 M Cacodylate Buffer for 10 mins. Blocking was performed by immersing spheroids in 1% Uranyl Acetate for 1 hour; spheroids were washed with distilled water and by dehydrated with 50%, 70%, 85%, 95% ethanol for 10 mins each, the 100% ethanol three times for 10 mins each. Spheroids were immersed 1× in 50:50 Ethanol:Propylene Oxide and 3× in Propylene Oxide for 10 mins each. Spheroids were then transferred to 50:50 Epon:Propylene Oxide for 3 hrs, then placed in a vacuum for 1 hr. The previous step was repeated with 80:20 Epon:Propylene Oxide mix then pure Epon (twice) for 3 hrs and in vacuum for 1 hr. Blocks were finally transferred to a 60°C oven overnight. Blocks were sectioned for Transmission Electron Microscopy and analysed using a JEOL JEM 2100 200 Kv Transmission Electron Microscope (JEOL, Peabody, MA).

### Gene expression microarray analysis

RNA was extracted from 2D or 4-day old 3D cultures using the Illustra RNAspin mini kit (GE Heathcare) and microarray analyses performed using the Illumina HT-12 Gene Expression Beadchips (Illumina, San Diego, CA, USA) at the USC Epigenome Centre core facility. Data have been deposited onto the GEO database. Raw data were analysed using methods from the specified Bioconductor [46] packages; beadarray to import and process the raw data from the chip images [47], the BASH algorithm [48] for detecting and managing spatial artefacts; the package limma [49], to implement background correction using negative control probes and quantile signal normalisation using negative and positive control probes [50]. Summary data was exported as log transformed mean values of probe signals. For differential gene expression analysis the log-transformed summary probe expression data were analysed using an implementation of the Signifcance Analysis of Microarrays (SAM) method [51] in the package siggenes. A two-class analysis using a modifed t-statistic was used to identify genes that were differentially expressed according to their culture conditions.

### Gene ontology analysis

The R package GOstats was used to identify gene ontology terms that are over/under represented in the differentially expressed genes. An implementation of the Hypergeometric test [52] was performed using the function hyperGTest. This computes Hypergeometric p-values for over or under-representation of each GO term in the specified ontology among the GO annotations for genes of interest. P-values were corrected for multiple testing of the total number of ontology terms, using the method described by Benjamini & Hochberg [53].

### Cluster analysis

Gene expression data for human fallopian tube epithelial cells were downloaded from the Gene Expression Omnibus (http://www.ncbi.nlm.nih.gov/geo/). The data of Tone *et al.* and George *et al*. [17,18] and were downloaded as raw files from GEO (GSE28044 and GSE12172). These data are profiles for microdissected fallopian tube epithelial cells thus minimizing the chance that contamination by stromal or immune cells could affect the profiles. Unsupervised hierarchical cluster analyses were performed to ascertain the quality of biological replicates and also how the relationships between cell lines and culture conditions impact upon gene expression, as well the similarities between culture conditions and primary tissue samples. Maximum and Euclidean distances were calculated, again in ‘R’, using Spearman’s or Pearson’s correlation on untransformed probe expression values and clustered by Ward’s minimum variance method. The data set supporting the results of this article is available in the GEO repository (http://www.ncbi.nlm.nih.gov/geo/), study identifier GSE51220.

## Abbreviations

FTSEC: Fallopian tube epithelial cell; polyHEMA: Poly-2-hydroxyethyl methacrylate; 3D: Three-dimensional; 2D: Two-dimensional.

## Competing interests

The authors declare that they have no competing interest.

## Authors’ contributions

KL and SAG designed the study and wrote the manuscript. KL, MN and NL performed the experiments. EB provided pathology expertise. CJ analyzed the microarray data. All authors read and approved the final manuscript.

## Supplementary Material

Additional file 1: Table S1 Gene expression microarray analysis of gene expression changes associated with transfer of fallopian tube secretory epithelial cells (FTSECs) from a 2D to a 3D microenvironment. The 100 most significantly changing genes in 3D cultured FTSECs compared to 2D cultured FTSECs. 53 genes were significantly upregulated, 47 genes were significantly downregulated. Click here for file

Additional file 2: Figure S1 Validation of genes identified as differentially expressed in 2D and 3D cultured FTSECs. We validated the top 3 up- and downregulated genes by qPCR. Click here for file

Additional file 3: Figure S2 Cluster analyses comparing genome-wide transcriptomic profiles of 2D and 3D cultured FTSECs to follicular and luteal phase fallopian tube epithelium. Each point on the graph indicates an individual microarray profile, technical replicates of cultured cells are shown by colored circles, open circles denoting 2D cultured cells and closed circles denoting 3D cultured FTSECs. Black open circles indicate follicular phase fallopian tube epithelial samples, closed circles indicate luteal phase fallopian tube epithelium. Each point represents an individual patient. All patients from Georges *et al*. and Tone *et al*. [17,18] datasets are shown. Clustering is consistent regardless of cluster method used (a) Euclidean Spearman, (b) maximum Pearson and (c) maximum Spearman. Click here for file
