# Supplementary figures and images for: In vitro three-dimensional modeling of fallopian tube secretory epithelial cells
Source: BMC Cell Biol. 2013 Sep 27;14:43. doi: 10.1186/1471-2121-14-43 (PMC3849984; doi:10.1186/1471-2121-14-43)

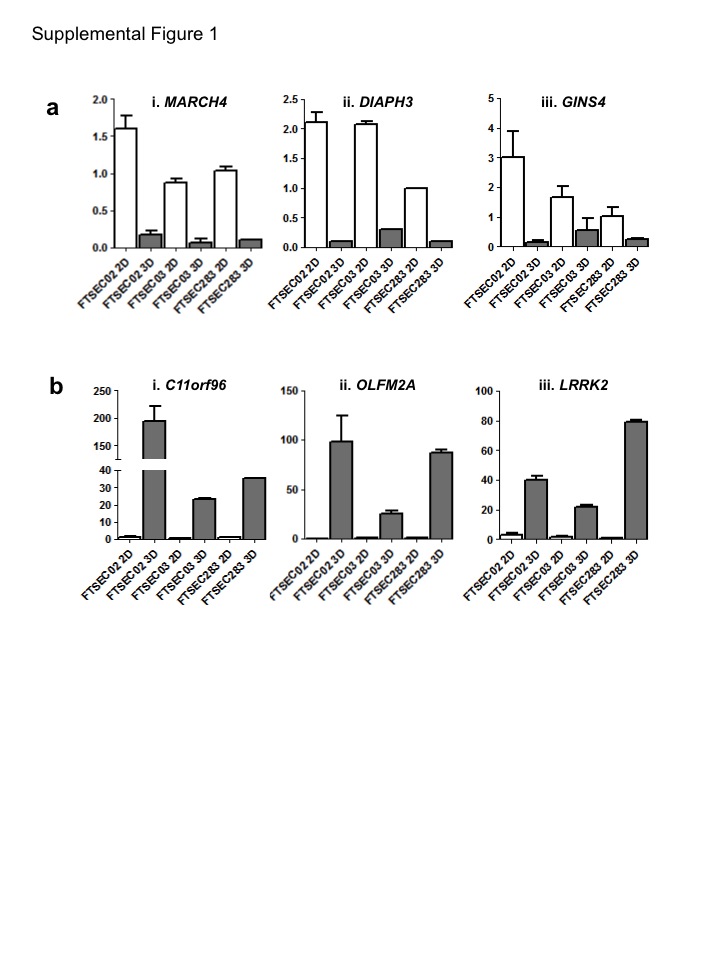

Supplement: Additional file 2: Figure S1 — Validation of genes identified as differentially expressed in 2D and 3D cultured FTSECs. We validated the top 3 up- and downregulated genes by qPCR. [file 1471-2121-14-43-S2.jpeg]

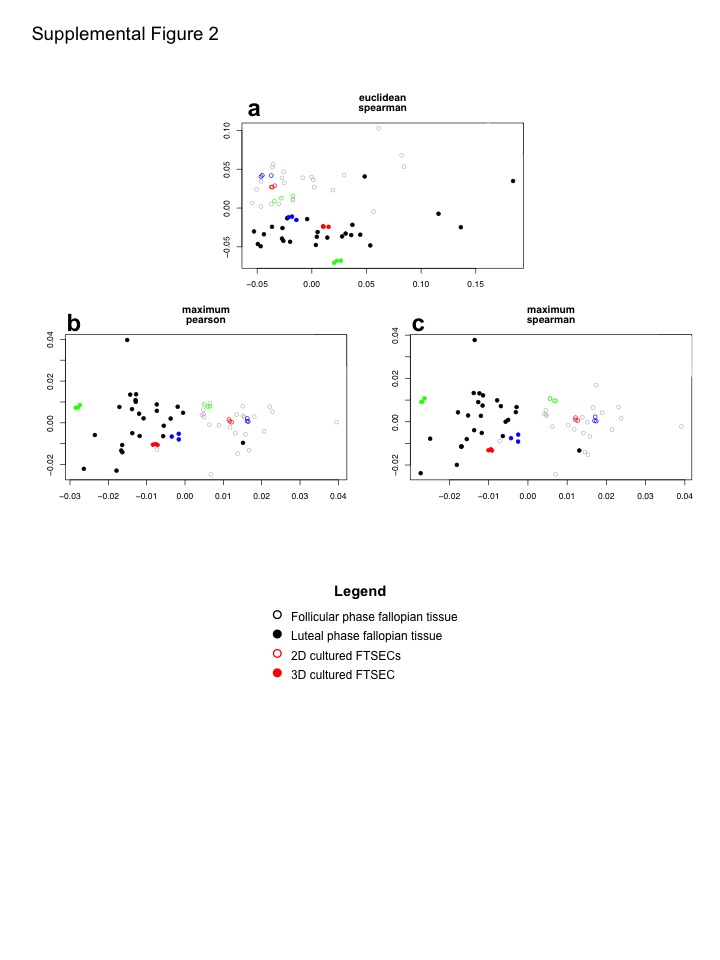

Supplement: Additional file 3: Figure S2 — Cluster analyses comparing genome-wide transcriptomic profiles of 2D and 3D cultured FTSECs to follicular and luteal phase fallopian tube epithelium. Each point on the graph indicates an individual microarray profile, technical replicates of cultured cells are shown by colored circles, open circles denoting 2D cultured cells and closed circles denoting 3D cultured FTSECs. Black open circles indicate follicular phase fallopian tube epithelial samples, closed circles indicate luteal phase fallopian tube epithelium. Each point represents an individual patient. All patients from Georges et al. and Tone et al. [17,18] datasets are shown. Clustering is consistent regardless of cluster method used (a) Euclidean Spearman, (b) maximum Pearson and (c) maximum Spearman. [file 1471-2121-14-43-S3.jpg]
